# Acceptability of Novel Formulated Ready to Use Food for Management of Moderate Acute Malnutrition (MAM) in Children Aged 6–59 Months in Tanzania: A Facility‐Based Acceptability Trial

**DOI:** 10.1002/fsn3.71093

**Published:** 2025-10-14

**Authors:** Kaunara Azizi, Hope Masanja, Nangida Jeska Mchome, Germana Leyna, Ray Masumo, Deborah Esau, Vera Lugutuah Kwara, Glory Benjamin, Erick Killel, Nyabasi Makori

**Affiliations:** ^1^ Department of Food Sciences and Nutrition Tanzania Food and Nutrition Centre Dar Es Salaam Tanzania; ^2^ Department of Community Health and Nutrition Tanzania Food and Nutrition Centre Dar Es Salaam Tanzania; ^3^ Department of Epidemiology and Biostatistics Muhimbili University of Health and Allied Sciences Tanzania; ^4^ Department of Mathematics and Statistics University of Dar Es Salaam Dar Es Salaam Tanzania; ^5^ World Food Programme Dar Es Salaam Tanzania; ^6^ Department of Nutrition Education and Training Tanzania Food and Nutrition Centre Dar Es Salaam Tanzania

**Keywords:** acceptability, children aged 6–59 months, moderate acute malnutrition, ready‐to‐use food products

## Abstract

Malnutrition contributes to nearly half of all deaths among children below 5 years of age; it is a significant burden of suboptimal development in children who survive. Novel Ready‐to‐Use Food Supplements (RUFs) have been developed to treat moderate acute malnutrition (MAM) in children aged 6–59 months in Tanzania. This study aimed to assess the acceptability of these new RUFs, which are made from locally sourced ingredients. A facility‐based acceptability trial was conducted to evaluate the new RUF formulations for MAM management. A total of 317 children aged 6–59 months were systematically randomly sampled and enrolled. Paired preference tests comparing the two novel RUFs (RUF 1 and RUF 5) were performed in Temeke Municipality, Tanzania. For the preference tests, mothers or caregivers received one 100 g sachet of either RUF 1 or RUF 5 daily for 2 days. For the acceptability trial, one sachet was provided every other day for 2 days. Each child tasted one of the novel RUFs on each day. Both novel RUFs demonstrated high acceptability, with over 90% of children accepting them. The mean average rating for RUF 5 was significantly higher than for RUF 1 across all attributes (*p* = 0.042). Mothers' or caregivers' liking rates for the porridge were 84.9% for RUF 1% and 87.7% for RUF 5 as MAM treatments. These findings underscore the importance of incorporating local taste preferences and cultural acceptability when developing therapeutic foods.

## Graphical abstract

The Ready to Use Food (RUF) product developed using the locally available food ingredients is acceptable among target population. It can be further assessed for its efficacy using randomized controlled trial.

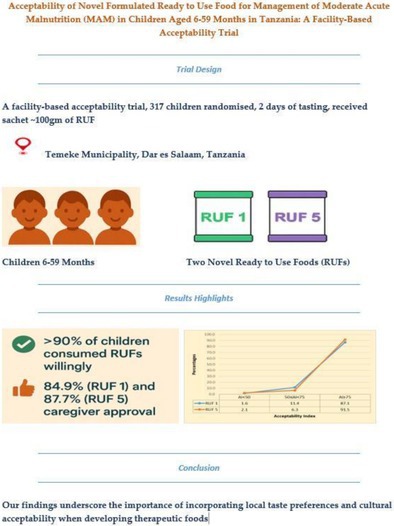

## 1 Introduction

Malnutrition affects over 45 million children under the age of 5 years globally, with over two‐thirds (31.4 million) suffering from moderate acute malnutrition (MAM) (WHO 2021). MAM is defined as a weight‐for‐height between −3 and −2 Z‐scores of the World Health Organization (WHO) child growth standards median and/or a mid‐upper‐arm circumference (MUAC) less than 125 mm (Actions 2020). Left untreated, MAM increases the risk of progression to severe acute malnutrition (SAM), which is associated with mortality, impaired cognitive development, and long‐term metabolic dysfunction (Dipasquale et al. 2020; Loechl 2014). Addressing MAM requires targeted nutritional interventions to reverse deficits in growth and development while preventing further deterioration.

Compared to normally nourished children, management of MAM requires a higher intake of nutrients and additional energy (Devi et al. 1985). WHO recommends a nutrient‐dense diet based on locally available foods rich in essential amino acids, healthy fats, vitamins, and minerals; these are important for improved health, development, and survival as well as for recovery in terms of weight and height (WHO 2023). Ready‐to‐use food (RUFs) products are widely known to have a fundamental role in supplementing dietary needs in malnutrition, hence preventing growth faltering in children (Borg et al. 2019). Ready‐to‐use therapeutic foods (RUTFs) have been recommended as home‐based therapy for treatment of uncomplicated SAM; likewise, RUSFs have also been developed for management of MAM (Osendarp et al. 2015). Ready‐to‐use food supplements have been proven to be more effective and easier to use as they do not require cooking compared to other commonly used supplementary foods such as fortified blended products, for example, corn soy bean porridge (CSB) (Borg et al. 2018).

Despite the proven benefits of RUFs, their local acceptability remains understudied. Many RUSFs rely on imported ingredients, which may not align with local dietary habits or become economically viable (Osendarp et al. 2015). Locally sourced RUFs could address these gaps by leveraging regionally available ingredients to improve affordability, sustainability, and cultural relevance (Borg et al. 2019). However, rigorous acceptability testing is critical before scaling, as even minor sensory mismatches can reduce compliance (Wanjiru Maina 2018).

To date, few studies have systematically evaluated the acceptability of novel, locally formulated RUFs for MAM management in children. This study fills this gap by assessing the acceptability of novel RUF formulation (ingredients; RUF 1: maize, soybean, and sesame seeds and RUF 5: maize, finger millet, soybean, and sesame seeds) among children aged 6–59 months and their caregivers, measuring consumption rate, sensory preferences, and caregiver willingness to adopt the product. The study also identified key determinants of acceptability (taste preference, aroma, color, and texture aversion) to inform product refinement.

## 2 Material and Methods

### 2.1 Product Development

Based on the local availability of food ingredients in Tanzania, the linear programming (LP) optimized model was made with optimal proportions of food ingredients based on the Excel solver in function to obtain blends of essential amino acid composition similar to that recommended by FAO/WHO/UNU for children with moderately malnourished children (Golden 2009; WHO/FAO/UNU Expert Consultation 2007). Two formulations were selected for an acceptability trial: RUF 1 (70% maize, 25% 5% sesame seeds, and 3% sugar) and RUF 5 (70% maize, 2% finger millet, 25% soybean, 3% sesame seeds, and 3% sugar).

### 2.2 Trial Design

This was a facility‐based acceptability trial, designed to investigate the acceptability of the two new formulations of RUFS for promoting growth and management of MAM in children aged 6–59 months.

### 2.3 Study Site and Population

The trial was conducted in 15 health facilities within Temeke Municipality in Dar es Salaam, chosen for its representation of urban poor communities with high rates of child wasting. Participants were infants and young children aged 6–59 months attending Reproductive and Child Health (RCH) at health facilities for usual nutritional screening clinics. Mothers/caregivers of selected children were the respondents. Children who had an acute illness or chronic disease, any congenital anomalies, participated in other nutrition interventions, or parental/guardian refusal to participate in the study were excluded from the study.

### 2.4 Sample Size Determination, Sampling Procedure, and Process

The study enrolled 317 children aged 6–59 months, a sample size determined to be sufficient for detecting between‐group differences in a two‐sided test with a Type I error rate of 0.05% and 90% power. This estimation was based on the assumption that the standard deviation of consumption would be 0.9% of the amount offered, meaning a coefficient of variation of 0.9. The objective was to achieve 90% power to reject the null hypothesis if the true means differed by at least 50%. A systematic random sampling approach was used to select and enroll children in the study to detect differences between groups. These participants were mobilized from the community and captured from their usual RCH clinic schedule for screening prior to enrollment.

The sample size calculation followed the formula below (Clifton et al. 2019):

$$\frac{n=2*{\left(Z_{1-\frac{\alpha }{2}}+Z_{1-\beta }\right)}^{2}*\sigma^{2}}{d^{2}}$$

where n is the sample size per group, *Z*_1_ − α/2 is the *Z*‐score for the significance level, *Z*_1_ − *β* is the *Z*‐score for the desired power, *σ* is the assumed standard deviation of consumption, and *d* is the minimum detectable difference in means.

### 2.5 Subject Recruitment

A mother–child pair was identified through RCH clinics; infants and young children were screened for nutritional status by trained enumerators. Upon fulfilling the enrolment criteria (age 6–59 months, started semi‐solid food) and receiving the consent for participation in the study from the parents or legal guardians, the children together with their respective mother/caregiver were enrolled and randomly allocated into two different study groups.

### 2.6 Description of Interventions

Participants assessed both RUF 1 and RUF 5 formulations in a crossover sensory evaluation, allowing within‐subject comparison of preference and acceptability. Mothers/caregivers received one sachet of approximately 100 g of one of the two novel RUFs per day for 2 days. In order to avoid bias among the enumerators, the sachets of the two products were coded with numerals and did not have any details on a particular product. Each child had an opportunity to taste the products, RUF1 and RUF2. After tasting the two products, enumerators asked mothers/caregivers about their preference for the tested sample products. Preference was determined through mothers receiving a 100 g RUF sachet, each containing 500 kcal, intended for children every other day for 6 days. Sachets were coded with numerals and without details to avoid bias among the enumerators. Each child had an opportunity to taste the two products through prepared porridge. Thereafter, a mother would choose one product based on the child's preference.

The RUFs were packaged in 1.7 kg foil bags, which equated to 14 days of rations. Each meal was to consist of 40 g of RUF (167 kcal), prepared by mixing with water in a ratio of 1:4 (RUF to water). Caregivers were advised to serve the porridge across three meals per day, ensuring each meal did not exceed 200 mL to accommodate the child's gastric capacity. Any leftovers were to be discarded according to the instructions provided. If a child could not finish a sachet in 1 day, it was discarded, and a new one was used the next day.

### 2.7 Outcomes Measurement

Acceptability of the food was defined as consuming 50% of the provided intervention within 15–30 min from the time it was first swallowed. Being able to consume 75% of feed was defined as a high acceptability, respectively. Parents/caregivers tasted food products before giving them to their children, which allowed them to predict the child's preference and determine the food acceptability to a child. Parents or guardians ranked the intervention as per preference. This was used to determine the acceptance of either intervention.

### 2.8 Data Collection

A pretested questionnaire was programmed into the Kobo Toolbox software. Parents/guardians identified formulas a child preferred in taste, color, odor, and texture based on a child's reaction. Questions were reported using a 9‐point hedonic scale, with 9 being the most preferable aspect of the RUF formula.

### 2.9 Statistical Analysis

All data were recorded in Excel and analyzed using SPSS software V.17. Continuous variables were analyzed using descriptive statistics (mean and standard deviation), while categorical variables were summarized as frequencies and percentages. The acceptability index, indicating the level of acceptance for each RUF product, was computed using the following formula (Gomes et al. 2015):

$$\text{Acceptability Index}\;\left(\%\right)=\frac{\text{Average score of product}}{\text{Maximum possible score}\times 100}$$

The analysis of variance (ANOVA) was applied to test the significance of differences in liking the attributes between formulation levels, with significance considered at *p* < 0.05.

## 3 Results

### 3.1 Demographic Characteristics of the Study Participants

A total of 317 children and their caregivers were enrolled in the acceptability trial. Table 1 summarizes the demographic characteristics of the participants. The children had a mean age of 20.1 months (SD = 12.5 months), with females comprising 51.2% of the participants. The majority of the caregivers were the child's biological mother (81%), with fathers (10%) and grandmothers (7%) making up the remaining caregiver roles. A significant proportion of mothers/caregivers (78.6%) were single. Over half of the respondents (58.7%) reported having primary education. A large proportion of mothers/caregivers were employed in the informal sector, with traders (41.5%) and housewives (36.4%) being the most prevalent occupations.

**TABLE 1:** Socio‐demographic characteristics of the study population.

Characteristic *N* = 317	Frequency	Percent
Age group (months)		
6–17	178	56.2
18–29	74	23.3
30–41	40	12.6
42–53	18	5.7
54–59	7	2.2
Sex of a child		
Male	154	48.58
Female	163	51.42
Education		
No education	7	2.21
Primary	186	58.68
Secondary and above	124	39.12
Marital status		
Married	58	18.3
Single	249	78.55
Separated/divorced	9	2.84
Widowed	1	0.32
Occupation		
Farming	13	4.11
Trading	131	41.46
Artisan	18	5.7
Pastoralist	2	0.63
Formal employment	11	3.48
Informal employment	11	3.48
House wife	115	36.39
Others (Specify)	15	4.75
Relation to the child		
Mother	293	92.43
Sister	5	1.58
Aunt	6	1.99
Grandmother	8	2.52
Others (specify)	5	1.58
Household use electricity		
No	257	81.07
Yes	60	18.93

### 3.2 Acceptability and Preference

**FIGURE 1:**
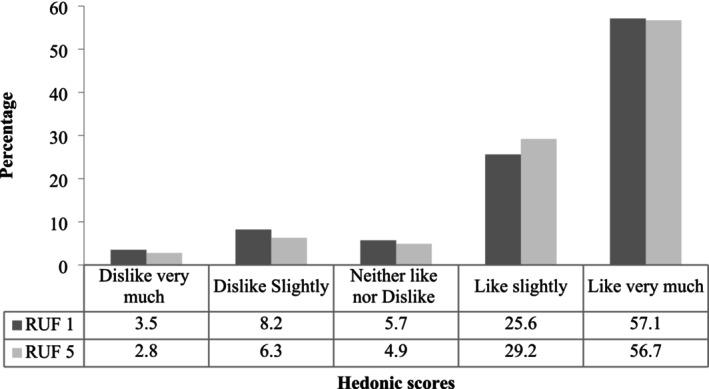
Amount of RUF formulated product consumed by a child in the form of porridge, *n* = 317.

**FIGURE 2:**
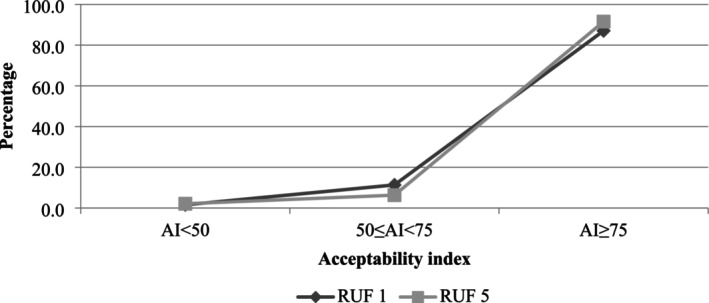
Percentage of respondents that indicated they “slightly liked”, “moderate liked,” or “strongly liked” either RUF‐formulated product using acceptability index, *n* = 317.

Children's consumption of both RUF products (RUF 1 and RUF 5) in terms of quality was comparable within 15–30 min of initial provision. Across the entire study population, 78% of children consumed half or more of the provided cup for both RUFs, with the majority (41.3% and 40.1% for RUF 1 and RUF 5, respectively) consuming a full cup (Figure 1). RUF 1 was highly acceptable to 87.1% while RUF 5 was even more acceptable to the children, 91.5% (Figure 2). Notably, only a small proportion of participants found either RUF formula unacceptable (1.6% and 2.1% for RUF 1 and RUF 5, respectively).

The mean average rating of each RUFs product based on each attribute score was significantly higher in the RUFs 5 than in RUFs 1 (*p* = 0.042). The sweetness and texture of RUF 5 porridge were significantly more preferred than that of RUF 1 porridge. Even though there was no statistical difference, the overall liking, color, taste, and smell of the RUFs 5 porridge were more preferred than that of RUFs 1 (Table 2).

**TABLE 2:** Acceptability scores for the RUF formulated product tested by mothers/caregivers in the form of porridge.

Properties	RUFs 1	RUFs 5	*p*
Overall liking	7.40 ± 2.09	7.54 ± 2.02	0.410
Color	8.44 ± 1.12	8.59 ± 1.16	0.124
Taste	8.06 ± 1.69	8.18 ± 1.66	0.356
Sweetness	7.56 ± 1.92	7.97 ± 1.61	0.005^**a**^
Texture	8.25 ± 1.40	8.49 ± 1.10	0.019^**a**^
Aroma	8.24 ± 1.38	8.30 ± 1.50	0.573
Average rating	7.99 ± 1.12	8.18 ± 1.13	0.042^**a**^

^a^ There is a significant difference *p* < 0.05.

**FIGURE 3:**
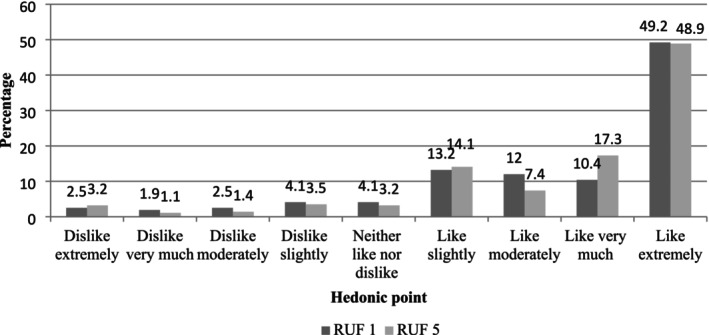
Mother/caregiver overall liking of the RUF formulated product, *n* = 317.

**FIGURE 4:**
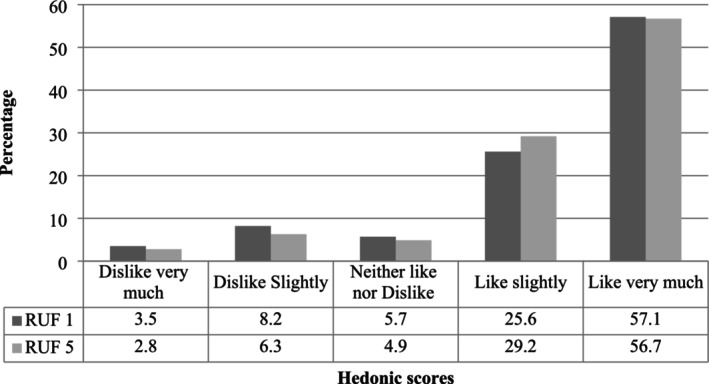
Mother/caregiver perception of child's overall liking of the RUF product formulated, *n* = 317.

Mother/caregiver's porridge liking rate was 84.9% for RUF 1% and 87.7% for RUF 5 (Figure 3). This is similar to the assessment of mother/caregiver's perception of children porridge liking rate, whereby RUF 5 was more preferred by the children (86%) than RUF 1 (83%) (Figure 4).

## 4 Discussion

Findings from the present study provide evidence that low cost, nutritious, and age‐appropriate complementary foods for young children can be locally developed and accepted among children under 5 years for treatment of MAM.

Different forms of food products have been developed for the purpose of addressing acute malnutrition among under‐five children in developing countries (Griswold et al. 2021; Iuel‐Brockdorf et al. 2015). However, the acceptability of the developed food products is more influenced by sensory quality than by nutritional quality and has a significant role in customers' emotional well‐being (Ramaroson Rakotosamimanana and De Kock 2020). The acceptability of ready‐to‐use therapeutic foods (RUFs) is a critical factor when addressing moderate acute malnutrition in young children, as their sustained consumption over a prescribed treatment period directly influences the success of the intervention. Our study tested two RUF formulations (RUF 1 and RUF 5) using sensory evaluation based on attributes such as color, taste, sweetness, texture, and aroma. The goal was to identify which product would be most suitable for efficacy trials.

Findings of the study show that both locally developed porridge flour for MAM treatment was highly acceptable by more than 90% (AI ≥ 75%) of children. A similar study conducted by Marchini et al. (2020), in the Rukwa region, confirmed that meals locally developed for complementary feeding were found culturally accepted and economically sustainable as they are made from locally available and economically accessible ingredients.

Both RUF formulations were generally accepted by the mothers/caregivers, with average scores above 7 of 9 across most attributes, indicating a high level of acceptance. However, certain sensory attributes, such as sweetness, texture, and overall average rating, were more favorable for RUF 5 compared to RUF 1. The appealing sensory profile of these formulations is crucial, particularly in young children who may refuse foods that are unfamiliar or unpleasant in texture and flavor (Boateng et al. 2018; Muhimbula et al. 2011). In addition to boosting the energy density of complementary food formulations, some previous studies reported that sugar addition improved the taste and distinctly improved flavor of the porridge, making them more palatable and appetizing than those without sugar (Konyole et al. 2012; Mkenya et al. 2013; Muhimbula et al. 2011). The total sugar present in millet might have contributed to the sweetness of the RUF 5 porridge in addition to the free sugar, which made the RUF 5 porridge sweetness score more acceptable (Dayakar et al. 2017). Also, even though the composite flour was milled, the incorporation of the whole millet grains contributed to the improved total dietary fiber content in RUF 5 flours. The presence of dietary fibers has mouthfeel‐related sensory impact, hence differentiating the RUF 5 porridge texture from RUF 1 (Chakraborty et al. 2019). The taste of both products was acceptable, with RUF 5 porridge tasting nonsignificantly more acceptable than RUF 1 porridge. The variation in acceptability is not statistically significant, suggesting that any preference for RUF 5 could be due to random chance rather than a difference in taste. Apart from the addition of sugar in both RUF products, the taste acceptance may also be attributed to the extrusion process. The extrusion process has an effect on taste, color, and aroma of food products. The application of heat to the composite flour during the extrusion process reduces sugar and amino acids that contribute to the formation of flavors, aromas, and colors (browning) preferred by most consumers (Verma et al. 2020). Processes involved to exhibit such effects include the Maillard reaction, caramelization, starch gelatinization, and dextrinization. However, since this was not evaluated in the current study, such interpretations should be viewed as speculative and are therefore limited in explanatory power.

A key strength of this study is its adequate sample size and facility‐based design, which allowed for a comprehensive assessment of the acceptability of two RUF formulations among mother–child pairs. Health facilities provided an appropriate setting, as they are the primary point of contact for most mothers and young children. The sufficient sample size also enabled the exploration of demographic differences that may influence acceptability. However, a limitation of the study is the short duration of the acceptability assessment, limited to 2 days, which does not allow for conclusions about long‐term adherence to the new formulations. Additionally, the study did not include cereal soy blend (CSB+) flour, as it is already an established and accepted product for the treatment of moderate acute malnutrition (MAM) among children under five in Tanzania. Nevertheless, the CSB+ was featured in the efficacy study while offering extended exposure to the RUF products, providing further insights into sustained acceptability over time.

The next phase of this research will involve an efficacy trial of RUF 5 to assess its potential to effectively treat moderate acute malnutrition in the target age group. Should the product demonstrate both efficacy and continued acceptability in the longer term, it could serve as a sustainable, locally produced solution for addressing malnutrition in Tanzania. Our findings highlight the importance of considering local taste preferences and cultural acceptability in the development of therapeutic foods. By utilizing locally sourced ingredients and tailoring the product to the sensory preferences of the community, we increase the likelihood of successful treatment outcomes and foster greater community ownership of malnutrition interventions.

## 5 Conclusion

Porridges prepared from both RUF formulations were well accepted by the target population, with no reported adverse effects. RUF 5 porridge was rated more favorably than RUF 1, particularly in terms of sweetness, texture, and overall acceptability. Although these findings suggest a preference for RUF 5, they are based on a short assessment duration, 2 days; hence, they should be interpreted with caution. It is noteworthy that all formulations received reasonably high scores across the tested parameters. These preliminary results provide useful insights and support further development of RUF 5 for evaluation in an efficacy trial, where its effectiveness in treating moderate acute malnutrition can be assessed more rigorously and over a longer period.

## Author Contributions

**Kaunara Azizi:** conceptualization (equal), formal analysis (equal), methodology (equal), writing – original draft (lead), writing – review and editing (equal). **Hope Masanja:** conceptualization (equal), formal analysis (equal), methodology (equal), writing – original draft (lead), writing – review and editing (equal). **Nangida Jeska Mchome:** data curation (equal), formal analysis (equal), writing – review and editing (equal). **Germana Leyna:** conceptualization (equal), methodology (equal), supervision (equal), writing – review and editing (equal). **Ray Masumo:** conceptualization (equal), methodology (equal), writing – review and editing (equal). **Deborah Esau:** writing – review and editing (equal). **Vera Lugutuah Kwara:** writing – review and editing (equal). **Glory Benjamin:** data curation (equal), formal analysis (equal), writing – review and editing (equal). **Erick Killel:** data curation (equal), formal analysis (equal), writing – review and editing (equal). **Nyabasi Makori:** conceptualization (equal), investigation (equal), supervision (lead), writing – review and editing (equal).

## Ethics Statement

The study protocol and data collection tools were approved by the National Health Research Ethics Sub‐Committee (NatHREC) of the National Institute of Medical Research (NIMR) of Tanzania, reference number NIMR/HQ/R.8a/Vol.IX/3630. Permission to conduct research in the respective municipal council was sought from the President's Office—Regional Administration and Local Government (PO‐RALG). The purpose of the study and methods of data collection, confidentiality, and voluntary participation were explained to the mothers/caregivers of children who were invited to participate in the study. Written informed consent was obtained from all caregivers of children who met the inclusion criteria before the recruitment of their children into the study. All interviews and intervention procedures were conducted in privacy.

## Conflicts of Interest

The authors declare no conflicts of interest.

## Data Availability

Data available on request due to privacy/ethical restrictions.

## References

[fsn371093-bib-0001] Actions, N. 2020. “E‐Library of Evidence for Nutrition Actions (eLENA) Supplementary Foods for the Management of Moderate acute Malnutrition in Children Aged 6–59 Months.”

[fsn371093-bib-0003] Boateng, L. , R. Nyarko , M. Asante , and M. Steiner‐Asiedu . 2018. “Acceptability of Complementary Foods That Incorporate *Moringa oleifera* Leaf Powder Among Infants and Their Caregivers.” Food and Nutrition Bulletin 39, no. 1: 137–148. 10.1177/0379572117708656. 28535743

[fsn371093-bib-0004] Borg, B. , S. Mihrshahi , M. Griffin , et al. 2018. “Randomised Controlled Trial to Test the Effectiveness of a Locally‐Produced Ready‐To‐Use Supplementary Food (RUSF) in Preventing Growth Faltering and Improving Micronutrient Status for Children Under Two Years in Cambodia: A Study Protocol.” Nutrition Journal 17, no. 1: 1–11. 10.1186/s12937-018-0346-x. 29548287 PMC5857085

[fsn371093-bib-0005] Borg, B. , S. Mihrshahi , M. Griffin , et al. 2019. “Acceptability of Locally‐Produced Ready‐To‐Use Supplementary Food (RUSF) for Children Under Two Years in Cambodia: A Cluster Randomised Trial.” Maternal & Child Nutrition 15, no. 3: 1–13. 10.1111/mcn.12780. PMC7198957 30690869

[fsn371093-bib-0006] Chakraborty, P. , T. Witt , D. Harris , J. Ashton , J. R. Stokes , and H. E. Smyth . 2019. “Texture and Mouthfeel Perceptions of a Model Beverage System Containing Soluble and Insoluble Oat Bran Fibres.” Food Research International 120: 62–72. 10.1016/j.foodres.2019.01.070. 31000279

[fsn371093-bib-0007] Clifton, L. , J. Birks , and D. A. Clifton . 2019. “Comparing Different Ways of Calculating Sample Size for Two Independent Means: A Worked Example.” Contemporary Clinical Trials Communications 13: 1–7. 10.1016/j.conctc.2018.100309. PMC6297128 30582068

[fsn371093-bib-0008] Dayakar, R. B. , K. Bhaskarachary , G. D. Arlene Christina , G. Sudha Devi , and A. T. Vilas . 2017. “Nutritional and Health Benefits of Millets.” In ICAR_Indian Institute of Millets Research (IIMR). CAB International. 10.1079/9781780648309.0024.

[fsn371093-bib-0009] Devi, C. D. S. , T. Ramesan , and G. Nath . 1985. “Technical Note.” International Journal of Heat and Mass Transfer 28, no. 10: 1960–1963. 10.1016/0017-9310(85)90220-0.

[fsn371093-bib-0011] Dipasquale, V. , U. Cucinotta , and C. Romano . 2020. “Acute Malnutrition in Children: Pathophysiology, Clinical Effects and Treatment.” Nutrients 12, no. 8: 1–9. 10.3390/nu12082413. PMC7469063 32806622

[fsn371093-bib-0012] Golden, M. H. 2009. “Proposed Recommended Nutrient Densities for Moderately Malnourished Children.” Food and Nutrition Bulletin 30, no. 3: S267–S342. 10.1177/15648265090303s302. 19998863

[fsn371093-bib-0013] Gomes, P. D. , F. L. F. Z. Sanches , E. F. D. Santos , M. R. Manhani , and D. Novello . 2015. “Carrot Cupcakes Development Added Flax Flour ( *Linum Usitatissimum* L.).” Physicochemical Composition, Sensory Acceptability Among Children and Relationships With Nutritional Status 7, no. 17: 78–93.

[fsn371093-bib-0014] Griswold, S. P. , B. K. Langlois , Y. Shen , et al. 2021. “Effectiveness and Cost‐Effectiveness of 4 Supplementary Foods for Treating Moderate Acute Malnutrition: Results From a Cluster‐Randomized Intervention Trial in Sierra Leone.” American Journal of Clinical Nutrition 114, no. 3: 973–985. 10.1093/ajcn/nqab140. 34020452 PMC8408853

[fsn371093-bib-0015] Iuel‐Brockdorf, A. S. , T. A. Dræbel , C. Fabiansen , et al. 2015. “Acceptability of New Formulations of Corn‐Soy Blends and Lipid‐Based Nutrient Supplements in Province Du Passoré, Burkina Faso.” Appetite 91: 278–286. 10.1016/j.appet.2015.04.058. 25913687

[fsn371093-bib-0016] Konyole, S. O. , J. N. Kinyuru , B. O. Owuor , et al. 2012. “Acceptability of Amaranth Grain‐Based Nutritious Complementary Foods With Dagaa Fish ( *Rastrineobola argentea* ) and Edible Termites (Macrotermes Subhylanus) Compared to Corn Soy Blend Plus Among Young Children/Mothers Dyads in Western Kenya.” Journal of Food Research 1, no. 3: 111. 10.5539/jfr.v1n3p111.

[fsn371093-bib-0017] Loechl, C. 2014. “International Symposium: Understanding Moderate Malnutrition in Children for Effective Interventions.” Sight and Life Magazine: Science and Implementation 2014, no. 3: 9–11. 10.52439/iarg6699.

[fsn371093-bib-0018] Marchini, M. , A. Rosi , F. Giopp , V. Lolli , F. Scazzina , and E. Carini . 2020. “The “Pappa di Parma” Integrated Approach Against Moderate Acute Malnutrition.” Innovative Food Science and Emerging Technologies 66: 102534. 10.1016/j.ifset.2020.102534.

[fsn371093-bib-0019] Mkenya, H. , L. Sharafadeen , and D. Aderomu . 2013. “Sensory Evaluation of Complement Food From Available Cereals and Legumes in Iringa, Tanzania.” Advances in Food Science and Technology 1, no. 5: 68–72.

[fsn371093-bib-0020] Muhimbula, H. S. , A. Issa‐Zacharia , and J. Kinabo . 2011. “Formulation and Sensory Evaluation of Complementary Foods From Local, Cheap and Readily Available Cereals and Legumes in Iringa, Tanzania.” African Journal of Food Science 5, no. 1: 26–31.

[fsn371093-bib-0021] Osendarp, S. , B. Rogers , K. Ryan , et al. 2015. “Ready‐To‐Use Foods for Management of Moderate Acute Malnutrition: Considerations for Scaling Up Production and Use in Programs.” Food and Nutrition Bulletin 36, no. 1: S59–S64. 10.1177/15648265150361S110. 25902616

[fsn371093-bib-0022] Ramaroson Rakotosamimanana, V. , and H. L. De Kock . 2020. “Sensory Studies With Low‐Income, Food‐Insecure Consumers.” Current Opinion in Food Science 33: 108–114. 10.1016/j.cofs.2020.03.010.

[fsn371093-bib-0023] Verma, V. , Z. Singh , and N. Yadav . 2020. Research Trends in Food Technology and Nutrition. AkiNik Publications. 10.22271/ed.book.905.

[fsn371093-bib-0024] Wanjiru Maina, J. 2018. “Analysis of the Factors That Determine Food Acceptability. *~253~* .” Pharma Innovation Journal 7, no. 5: 253–257.

[fsn371093-bib-0025] WHO . 2021. UNICEF / WHO / the World Bank Group Joint Child Malnutrition Estimates. Global Health Observatory.

[fsn371093-bib-0026] WHO, W. H. O . 2023. “WHO Guideline on the Prevention and Management of Wasting and Nutritional Oedema (Acute Malnutrition) in Infants and Children Under 5 Years.” 38498638

[fsn371093-bib-0027] WHO/FAO/UNU Expert Consultation . 2007. “Protein and Amino Acid Requirements of Infants and Children.” In Nutrition Abstracts and Reviews, vol. 935. National Academies Press.

